# Long‐Term Cardiovascular Outcomes Following New‐Onset Arrhythmias During Pregnancy

**DOI:** 10.1111/pace.70283

**Published:** 2026-05-12

**Authors:** Sanya Dhama, Yong Hao Yeo, Mohammed Uddin, Rhea Dhama, Kamala P. Tamirisa

**Affiliations:** ^1^ School of Medicine University of California‐Riverside Riverside California USA; ^2^ Department of Internal Medicine and Pediatrics Corewell Health William Beaumont University Hospital Royal Oak Michigan USA; ^3^ University of California San Diego San Diego California USA; ^4^ Department of Cardiovascular Medicine University of Texas Southwestern Medical Center Dallas Texas USA

**Keywords:** arrhythmia, cardiovascular outcomes, pregnancy

## Abstract

**Background:**

Arrhythmias during pregnancy are increasingly prevalent and associated with acute adverse cardiovascular outcomes, yet their long‐term cardiovascular implications remain poorly defined. To address this gap, we conducted a large population‐based study to evaluate 5‐year cardiovascular outcomes following incident arrhythmias during pregnancy.

**Methods:**

Using TriNetX, we conducted a retrospective cohort study of pregnant individuals (≥18 years) admitted for delivery between 2015 and 2020. Patients with new‐onset arrhythmias during pregnancy were compared with those without arrhythmias. Outcomes included composite cardiovascular outcomes, incident heart failure (HF), cerebrovascular accident (CVA), and acute myocardial infarction (AMI). Propensity score matching (1:1) was used to adjust for demographic and clinical comorbidities.

**Results:**

Among 886,732 deliveries, 8507 patients (0.9%) had incident arrhythmias. After matching, individuals with incident arrhythmias during pregnancy had higher 5‐year risks of composite cardiovascular outcomes (risk ratios [RR] 3.82), HF (RR 4.48), CVA (RR 2.04), and AMI (RR 4.80) (all *P* < 0.01). Associations persisted across age strata, with particularly elevated risks among those ≥35 years.

**Conclusions:**

This study, among the first to our knowledge to evaluate long‐term cardiovascular outcomes following incident arrhythmias during pregnancy, demonstrates a substantially increased 5‐year cardiovascular risk, underscoring the need for extended postpartum cardiovascular surveillance beyond routine follow‐up.

## 1 Introduction

Arrhythmias during pregnancy are increasingly prevalent, with pregnancy‐related hospitalizations involving arrhythmias increasing by 58% over 10 years [1]. They have been associated with adverse short‐term maternal and fetal outcomes, including increased maternal mortality during hospitalization for delivery [1, 2]. However, data on long‐term cardiovascular outcomes after pregnancy‐associated arrhythmias remain limited. To address this gap, we conducted a population‐based retrospective cohort study evaluating 5‐year cardiovascular risk following new‐onset arrhythmias during pregnancy.

## 2 Methods

This retrospective cohort study used TriNetX, a global health research network containing de‐identified records from over 100 million patients. We identified pregnant individuals (≥18 years) admitted for delivery (ICD‐10 O80–O82) from January 2015 to December 2020. Patients were stratified by the diagnosis of new‐onset arrhythmias during pregnancy, including supraventricular tachycardia (SVT; I47.1), atrial fibrillation/flutter (AF/AFL; I48), ventricular tachyarrhythmias (I47.0, I47.2, I49.0), and unspecified cardiac arrhythmias (I49.8, I49.9). Patients with any documented diagnosis of arrhythmias, heart failure (HF) (I50), ischemic heart disease (I20–I25), or cerebrovascular accident (CVA) (I63) before pregnancy were excluded to restrict the cohort to incident arrhythmias during pregnancy. Outcomes were assessed from the time of delivery through 5 years, with only the first event counted. Outcomes included incident HF, CVA, and acute myocardial infarction (AMI) (I21–I24). We also assessed a composite cardiovascular outcome for each group, defined as the occurrence of HF, CVA, or AMI. Prespecified subgroup analyses stratified patients by maternal age (<35 vs. ≥35 years), comparing the risks of long‐term outcomes between those with and without arrhythmias within each age stratum. Propensity score matching (PSM) (1:1) was performed, adjusting for demographic and medical/obstetric comorbidities (diabetes mellitus, hypertensive disorders, adult congenital heart disease, and renal disease), with standardized mean differences <0.1 indicating adequate covariate balance. Risk ratios and *p* values were reported. Institutional review board approval was not required due to the use of de‐identified data.

## 3 Results

We identified 886,732 patients admitted for delivery: 8507 with incident arrhythmias (0.9%; age 28.9 ± 7.3 years) and 878,225 without (91.9%; age 28.8 ± 6.1 years). Following PSM, 8505 patients were included in each group with well‐balanced baseline characteristics. Among patients with specified types of tachyarrhythmias (*n* = 3129), 2084 (66.6%) had SVT, 604 (19.3%) had AF/AFL, and 441 (14.1%) had ventricular arrhythmias. At the 5‐year follow‐up, those with incident arrhythmias had a higher risk of composite cardiovascular outcomes (3.4% vs. 0.9%; RR 3.82; 95% CI 2.97–4.90; *P* < 0.01) compared with those without arrhythmias. They were also associated with higher risks of individual cardiovascular outcomes: HF (2.3% vs. 0.5%; RR 4.48, 95% CI: 3.23–6.20; *P* < 0.01), CVA (0.6% vs. 0.3%; RR 2.04, 95% CI: 1.27–3.29; *P* < 0.01), and AMI (0.8% vs. 0.2%; RR 4.80, 95% CI: 2.75–8.37; *P* < 0.01). In age‐stratified analyses (Figure 1), the association between incident arrhythmias and 5‐year composite cardiovascular outcomes and HF persisted in both age groups (<35: composite cardiovascular outcomes RR 5.22, 95% CI: 3.16–8.63; *P* < 0.01, HF RR 4.47, 95% CI: 2.56–7.80; *P* < 0.01; ≥35: composite cardiovascular outcomes RR 3.77, 95% CI: 2.78–5.10; *P* < 0.01, HF RR 3.82, 95% CI: 2.63–5.57; *P* < 0.01). The association between incident arrhythmias and 5‐year CVA and AMI was significant among those ≥35 years (CVA RR 2.44, 95% CI: 1.36–4.36; *P* < 0.01, AMI RR 2.78, 95% CI: 1.62–4.75; *P* < 0.01) but was not assessed among those <35 years due to the small number of events (<10).

**FIGURE 1:**
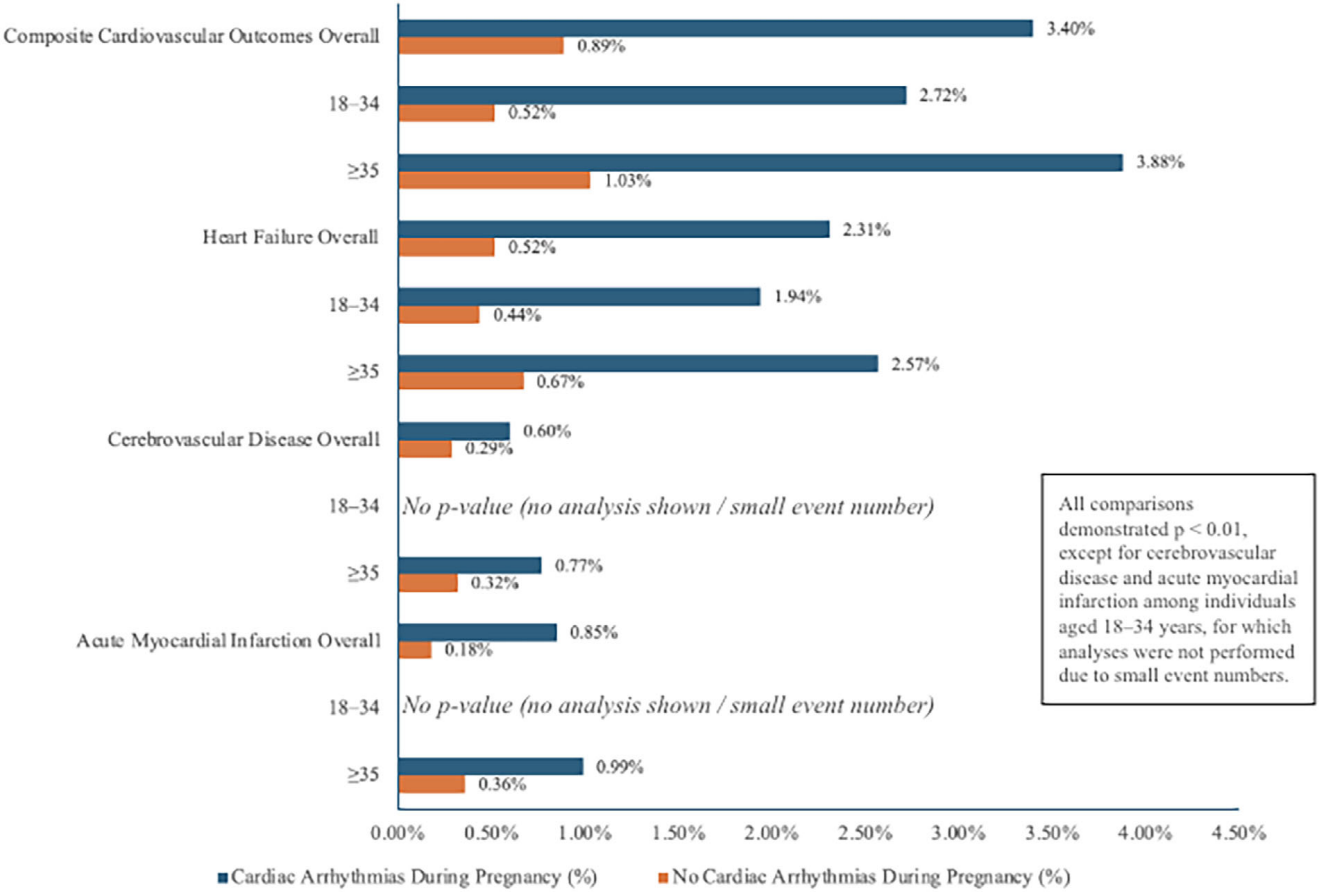
Long‐term cardiovascular outcomes of patients with incident cardiac arrhythmias during pregnancy. [Colour figure can be viewed at wileyonlinelibrary.com]

## 4 Discussion

In this population‐based cohort of individuals with new‐onset arrhythmias during pregnancy, long‐term cardiovascular event rates ranged from approximately 0.6% to 3.9%, substantially lower than the cardiac event rates reported in modified World Health Organization (mWHO) risk classes (9.9%–28.9%) [3]. This likely reflects differences in case mix, as our cohort captured incident arrhythmias occurring during pregnancy only, rather than individuals with pre‐existing cardiovascular disease. Importantly, this study evaluated outcomes beyond traditional obstetric risk stratification periods, including the postpartum window of maximal physiologic cardiovascular stress (first 6 weeks extending through one year), a critical time for cardiovascular risk assessment and intervention [4, 5]. HF emerged as the most frequent long‐term outcome, consistent with prior evidence linking arrhythmias during hemodynamic stress of pregnancy with adverse ventricular remodeling and cardiomyopathy, though causality cannot be inferred [6, 7]. Although age ≥35 years was associated with higher absolute event rates, individuals <35 years demonstrated comparable relative risk, suggesting that age alone may not explain postpartum cardiovascular vulnerability or long‐term cardiovascular risk. These findings support pregnancy as a physiologic stress test and highlight the need for longitudinal risk surveillance across age strata [8].

This study has several limitations. As with any database‐dependent analysis, results relied solely on accurate coding. Arrhythmia subtypes were aggregated due to small subgroup sizes and database restrictions on reporting low‐frequency events for patient confidentiality. Although PSM was robust, residual confounding from unmeasured factors, such as parity, socioeconomic status, or arrhythmia management, may remain. Given the retrospective and observational design, this study does not establish causality between incident arrhythmias in pregnancy and long‐term cardiovascular outcomes.

Despite these limitations, our findings suggest a substantially increased long‐term cardiovascular risk among individuals with incident arrhythmias during pregnancy.

## 5 Conclusion

To our knowledge, this is among the first large, population‐based studies linking incident arrhythmias during pregnancy to adverse cardiovascular outcomes extending five years postpartum. Prospective studies are needed to validate these associations and to identify clinical and biologic factors that can refine risk stratification and guide targeted longitudinal surveillance beyond routine 6‐week follow‐up, especially among individuals with incident cardiac arrhythmias during pregnancy.

## Author Contributions

**Sanya Dhama**: conceptualization, data curation, formal analysis, investigation, methodology, project administration, visualization, writing – original draft. **Yong Hao Yeo**: conceptualization, investigation, methodology, project administration, supervision, validation, visualization, writing – original draft, writing – review and editing. **Mohammed Uddin**: data curation, formal analysis, methodology, project administration. **Rhea Dhama**: writing – original draft. **Kamala P. Tamirisa**: conceptualization, investigation, methodology, supervision, validation, visualization, writing – original draft, writing – review and editing.

## Funding

This research did not receive any specific grant from funding agencies in the public, commercial, or not‐for‐profit sectors.

## Ethics Statement

Ethics approval and patient consents were waived for this study because only aggregated deidentified data were analyzed and reported.

## Conflicts of Interest

Kamala P. Tamirisa is a speaker for Boston Scientific and Zoll Medical. Rest of the authors declared that they have no affiliations with or involvement in any organization or entity with any financial interest (such as honoraria; educational grants; participation in speakers’ bureaus; membership, employment, consultancies, stock ownership, or other equity interest; and expert testimony or patent‐licensing arrangements), or non‐financial interest (such as personal or professional relationships, affiliations, knowledge or beliefs) in the subject matter or materials discussed in this manuscript.

## Data Availability

The processed data required to reproduce the above findings cannot be shared at this time due to technical limitations.
